# Insomnia severity and multidimensional psychological well-being in Chilean correctional personnel: a cross-sectional study

**DOI:** 10.3389/fpubh.2026.1782308

**Published:** 2026-10-26

**Authors:** Isabel Benjumeda Wynhoven, Santiago Varela Sepúlveda, Ricardo Bastián Bravo Lara, Gastón Ocampo Vélez

**Affiliations:** 1 Departamento de Ciencias, Facultad de Artes Liberales, Universidad Adolfo Ibáñez, Vina del Mar, Chile; 2 Escuela de Negocios, Universidad Adolfo Ibáñez, Vina del Mar, Chile; 3 Psicólogo de personal, área de salud, Gendamería de Chile, Valparaíso, Chile

**Keywords:** insomnia severity, prison health, prison workers, psychological well-being, subjective sleep quality

## Abstract

**Introduction:**

Prison officers in Chile work under highly stressful and dangerous conditions characterized by prison overcrowding, violence, long shifts, and chronic exposure to stress. Shift work and irregular schedules negatively affect sleep quality and contribute to insomnia, which in turn may impair psychological well-being. Despite growing evidence on occupational stress in correctional staff, limited research has examined the relationship between sleep quality, insomnia, and psychological well-being in prison guards. Therefore, this study aimed to assess working conditions, subjective sleep quality, insomnia severity, and psychological well-being among Chilean prison officers.

**Methods:**

A cross-sectional descriptive study was conducted with 43 prison guards from five prisons in the Valparaíso Region, Chile. Instruments included the Insomnia Severity Index (ISI), self-ratings of subjective sleep quality and the Ryff Psychological Wellbeing Scale (PWB). Sociodemographic and work-related variables were also analyzed, including sick leave, exposure to violence at work, need for mental health care, perceived organizational support, and energy drink consumption.

**Results:**

Participants reported low institutional support (60.5%) and frequent sick leave (68.4%). Over one-third considered seeking psychological care (37.2%), and 53.5% reported daily energy drink consumption. Insomnia levels were moderate and clinically relevant (M = 17.87, SD = 0.91), with high dissatisfaction and functional impairment. Psychological well-being was moderate overall (M = 54.84, SD = 16.52), with higher scores in positive relationships and lower scores in environmental mastery and personal growth. No significant association was found between ISI scores and overall or subscale psychological well-being. However, insomnia was positively associated with years at the institution (*p* = 0.023, *r* = 0.349), indicating that longer tenure relates to higher subjective insomnia severity scores.

**Conclusions:**

Chilean prison officers exhibited clinically significant insomnia, moderate psychological well-being, and low perceived institutional support. Although no significant association was found between insomnia and psychological well-being, insomnia severity increased with years of service. These findings underscore the need to improve working conditions and implement policies that promote occupational health in Chilean prisons. Incorporating a gender perspective in this highly masculinized context is also recommended, as well as further research to enhance psychological well-being.

## Introduction

Latin American prisons are considered the most dangerous in the world due to severe overcrowding, violence and human rights violations (1–3). Prison officers are responsible for ensuring the efficient operations of penitentiary systems and are exposed to a constant threat to their well-being (4, 5). These officers are also affected by conditions of deprivation of liberty, together with being exposed to high levels of stress and violence (6, 7). This may lead to burnout, post-traumatic stress disorder (PTSD), depression, anxiety, suicide, sleep problems and chronic pain (7–9). Around 35% of prison officers show major depressive disorder and general anxiety (10), and their suicide rate is 39% higher than that of general population (11).

In Chile, the mental health of prison officers is a critical issue, as they face extreme working conditions of stress and emotional risk, excessive workload, poor working conditions (7) and prison overcrowding (12–14).

Prison officers in Chile belong to Gendarmería de Chile, the national prison and correctional service, responsible for the administration and security of the country's penitentiary system. The first stage of a prison guards career in Chile begins with the post of prison guard, also known as an “armed guard” (up to 3 years) corresponds to guards working at the guard posts. During this phase, the guards are tasked with patrolling the perimeter of the prison guard posts, armed with handguns (service pistols) and shotguns. They also wear protective equipment such as bulletproof vests and helmets. After this period, the guards come down from the post and carry out surveillance duties within the prison complex, also performing other duties such as accompanying inmates to the hospital, which can lead to longer working hours, extending their shift. Shifts are normally 4 h long with a 4-h break, but they can be extended to 12 or 15 h, depending on the number of staff available and the requirements within the prison. Guard posts do not have toilets or chairs, to prevent guards from falling asleep. During their shift, guards are not allowed to leave their posts, either to go to the toilet or to get something to eat. Prison guards usually live inside the prison in specific areas designed for them, sharing rooms. Shift work, a defining characteristic of correctional settings, has been consistently associated with sleep disturbances, circadian misalignment, and increased fatigue among armed guard. Irregular schedules and night shifts have been shown to negatively impact sleep quality and increase the prevalence of insomnia-related symptoms in this population (15, 16). Given that sleep is a key determinant of psychological functioning, these disruptions may have important implications for psychological well-being, underscoring the need to examine sleep-related factors within occupational health research in correctional environments. Sleep disturbances are associated with different mental health disturbances and appear to be risk factors for suicidal ideation and planning (17, 18). Sleep disturbances encompass a broad range of sleep-related problems, including poor sleep quality and insomnia symptoms. Poor sleep quality refers to an individual's subjective perception of insufficient or non-restorative sleep, whereas insomnia involves difficulties initiating or maintaining sleep accompanied by daytime impairment (19). Both have been identified as common concerns in occupational settings and are associated with adverse mental health outcomes (20, 21).

Sleep disturbances among correctional officers should be understood within the broader context of safety-critical occupations characterized by shift work, high psychological demands, and sustained exposure to occupational stressors. Sleep health is increasingly recognized as a key determinant of occupational functioning, emotional regulation, and psychological well-being, as inadequate sleep may both result from and contribute to increased stress and reduced adaptive capacity. Recent evidence from high-demand academic settings indicates that elevated perceived stress and anxiety are associated with poorer sleep hygiene and reduced sleep quality, highlighting the bidirectional relationship between psychosocial demands and sleep-related outcomes (22). Similar associations have been identified in other safety-critical shift-working populations, such as security personnel and paramedics, where insomnia and poor sleep quality have been linked to lower work ability, impaired occupational functioning, and increased vulnerability to health problems (23). Moreover, research among night-shift technical workers suggests that chronic fatigue associated with disrupted sleep patterns may extend beyond psychological consequences, contributing to physical symptoms, including musculoskeletal complaints, and compromising recovery processes necessary for maintaining functional capacity (24). Together, these findings support the view that sleep disturbances in demanding occupational contexts are not isolated symptoms but reflect complex interactions between work conditions, psychological stress, and individual recovery processes.

Alterations in sleep and chronic stress are a diagnostic criterion for several disorders such as depression and burnout in prison guards (25–27). These disruptions to the sleep cycle can affect the quality of life and psychological well-being of prison guards, understood as a multidimensional construct reflecting optimal psychological functioning and adaptive human development. Within a eudaimonic framework, it encplantompasses key dimensions including autonomy, environmental mastery, personal growth, positive relationships, purpose in life, and self-acceptance, which together capture individuals' capacity to realize their potential and effectively manage life demands (28, 29).

This study addresses an important gap in occupational health research by examining the relationship between working conditions, sleep quality, insomnia severity and psychological well-being among prison guards in Chile, a population that remains underrepresented in literature. While prior research showed elevated levels of stress, burnout, and mental health difficulties in correctional staff, few studies have simultaneously considered sleep as a factor linked to well-being in this context. Given the unique demands of prison work, including shift schedules, exposure to chronic stress, and high-risk environments, understanding how sleep quality interacts with psychological functioning is both timely and necessary in this population. By integrating these dimensions, the present study offers a novel contribution to public health by highlighting potential pathways for intervention aimed at improving the overall well-being and occupational health of correctional personnel.

Accordingly, the present study aimed to examine the sociodemographic and working conditions of armed guard, including exposure to workplace violence and perceived institutional support, as well as to assess their psychological well-being, subjective sleep quality and insomnia severity levels. Additionally, the study explored the relationship between all these variables.

## Methodology

### Procedure and study design

Data for the current study was obtained from 5 different correctional institutions from the region of Valparaiso, Chile (Valparaiso, Limache, Quillota, Los Andes and San Antonio). We collected cross-sectional data through a survey distributed to the armed officers from January 2025 to February 2025. We invited prison guards to participate through personal invitations and data was collected individually through a self-administered survey. Each participant completed the questionnaires independently in a private, quiet environment to ensure confidentiality and minimize potential distractions or social influence during the response process. No external distractors or interruptions were present during data collection that could have influenced their responses.

All instruments used in this study were self-report measures, and participants provided their own responses based on their personal perceptions and experiences.

A total of 43 officers participated in the data collection (18 from Valparaíso, 6 from Limache, 7 from Quillota, 7 from San Antonio and 5 from los Andes). The current study included data only from participants who completed the sections on sleep quality and subjective well-being. Prior to answering the questions, participants signed informed consent, in which confidentiality, data storage, risks and benefits, and withdrawal of consent procedures were stated. Participants were informed that their data would be processed anonymously and confidentially and they all signed an informed consent.

Ethical approval was obtained from the Universidad Adolfo Ibáñez Ethical Committee prior to data acquisition. Data collection was performed from January 15^th^, 2025, to February 25^th^, 2025. The study participants were also informed that they could withdraw at any time without giving any reason for doing so. Consideration was also given to retribution to participants and the studied institution, through the delivery of a final research report to them.

### Sample

In this study, most of the sample (*N* = 30) had worked at the prison for between 1 and 3 years. The remainder (*N* = 9) had been in service for between 3 and 6 years, and a small proportion of the sample (*N* = 4) had been there for 10 years or more.

As previously described, the first track of prison guards (up to 3 years) corresponds to guards working at the guard boxes. After this period, the guards come down from the sentry boxes and carry out surveillance duties within the prison complex, also performing other duties such as accompanying inmates to the hospital, working in shifts. Prison guards usually live inside the prison in specific areas designed for them, sharing rooms.

Although the sample size is small (*N* = 43), it represents 33.8% of all prison guards of the five correctional facilities included in the study (*N* = 127), constituting a significant proportion within an institutional context of restricted access. Moreover, it should be noted that prison guards typically have high rates of absenteeism due to sick leave, which reduces the effective operational staff at the time of data collection. In this context, the sample obtained corresponds to a significant fraction of the staff that was available at the time of the study.

Furthermore, participants were recruited from five different correctional facilities in the Valparaíso Region, which provides institutional diversity and reduces the risk that the results reflect dynamics specific to a single facility. Given that prison guards perform highly standardized duties (guards work under a structured shift system), working conditions tend to be consistent across facilities, which supports the representativeness of the sample with respect to this segment of prison staff.

The inclusion criteria were part of the group of guards who work and live within prison grounds, carrying out mainly security duties and being on active duty at the time of data collection. The exclusion criteria were guards who were on medical leave at the time of the study.

### Instruments

Research has shown that the insomnia severity index (ISI) is a valuable tool for studying sleep disorders in correctional guards (26), whose unique work environment exposes them to chronic stress and irregular schedules (27). On the other hand, the Ryff Psychological WellBeing Scale (PWB) can be an effective tool for measuring psychological well-being in prisons, offering valuable insights into inmates' emotional and mental health (30).

### Insomnia severity

The Insomnia Severity Index (ISI) is a “a brief self-report instrument measuring the patient's perception of his or her insomnia [...] The ISI targets the subjective symptoms and consequences of insomnia as well as the degree of concerns or distress caused by those difficulties. Its content corresponds in part to the diagnostic criteria of insomnia” [(31), p. 602]. It contains 7 items and each item was rated on a 5-point Likert Scale ranging from 0 (very satisfied) to 4 (very dissatisfied). The ISI has good internal consistency, discriminatory capacity, and convergent validity in both community and clinical samples (21, 31) as well as in armed guard (26). According to the scoring ISI guidelines, a total score of 0–7 indicative no clinically significant insomnia, 8–14 indicate subthreshold insomnia and 15 and above moderate to severe clinical insomnia (21). It is important to note that, although the original ISI used a 2-week reference period (21), in the present study—following the article by Ricciardelli et al. (26), which was conducted with a similar sample in a prison setting—the reference period was 1 month, as previously stated by Bastien et al. (21), p. 302 (“The ISI is a 7-item self-report questionnaire assessing the nature, severity, and impact of insomnia. The usual recall period is the “last month” and the dimensions evaluated are severity of sleep onset, sleep maintenance, and early morning awakening problems, sleep dissatisfaction, interference of sleep difficulties with daytime functioning, noticeability of sleep problems by others, and distress caused by the sleep difficulties”). A Spanish validated version of the ISI was used (32).

### Sleep quality

As in the study conducted by Ricciardelli et al. (26), this research also included “four single items designed to assess sleep patterns and quality in the last month, including: (1) total hours of sleep on work nights (i.e., on average, approximately how many hours per night do you sleep, excluding time spent in bed not sleeping, on weeknights, or on-shift periods if you work shift work?); (2) total number of hours of sleep on off-shift nights (i.e., on average, approximately how many hours per night do you sleep, excluding time spent in bed not sleeping, on weekends, or off-shift periods if you work shift work?); (3) number of days per week you feel rested (i.e., on average, how many days per 7 day week do you wake up feeling rested?); and (4) an overall assessment of the quality of sleep (i.e., how would you rate your quality of sleep overall?) rated on a 5-point Likert Scale ranging from 0 (very poor) to 4 (very good)”. The same items as in Ricciardelli study were used [(26), p.3].

### Psychological well-being

On the other hand, the Ryff Scale of Psychological WellBeing (PWB) provides a multidimensional measure of well-being based on six core dimensions: Autonomy, Environmental Mastery, Personal Growth, Positive Relations with Others, Purpose in Life, and Self-Acceptance (33). For this study, we used the abbreviated 18-item version (28), offering a succinct yet comprehensive assessment of each dimension. Respondents rated items on a 7-point Likert scale ranging from 1 (strongly disagree) to 7 (strongly agree). Higher scores indicate greater levels of well-being across respective dimensions (15 to 21 points). Lower scores were from 1 to 7 and medium scores from 8 to 14. This version of the Ryff Scale has been validated for reliability and has shown consistent psychometric properties in various populations (28). The 18-item scale has also been used with Latino college students using a Spanish validated version (34), African Americans living in New York, and Mexican Americans living in Chicago (29). The Ryff Scale's use in prisons often reveals critical links between well-being and mental health interventions, suggesting the potential benefits of targeted support programs to improve personal growth and environmental mastery in prisons. These insights support integrating the Ryff Scale as a diagnostic and evaluative tool for mental health initiatives within correctional facilities, as seen in multiple studies examining well-being and social connectedness in the prison populations [(30, 35), in Spanish].

### Sociodemographic and occupational variables

Sociodemographic covariates analyzed included: sex (i.e., male or female), age, marital status (i.e., married, single, living together, separated/divorced/widowed), highest level of education (i.e., high school or less, some post-secondary [less than 4 year college/university program], and university degree of 4 year college program or higher). On the other hand, labor-related variables included total years of service at the institution, amount of medical leaves in the last year and the reason for it, psychiatric or psychological support requirements, medication use, consumption of energy drinks, have experienced violence at work in the past year, whether from colleagues or from prisoners, social support (if they had someone to call when they needed to) and feeling cared for by the institution. Participants reporting not feeling cared for by the institution were asked how this could be solved.

### Data collection process

Data collection was conducted on an individual basis with each guard between January and February 2025. The psychologist in charge either went up to the guard post or met with the guard in his office, where the guard completed the informed consent form, answered sociodemographic and work-related questions, and filled out the two questionnaires (ISI and Ryff) using pen and paper, due to the lack of internet access at the prison facility. On average, the total time to complete the instruments was between 20 and 25 min. The psychologist always remained with the guard, available to answer any questions or address any concerns during the process. Participants were informed that their data would be processed anonymously and confidentially and they all signed an informed consent.

### Data analysis

Data was analyzed using the Jamovi 2.3.21 software. Descriptive analysis was used to characterize the studied population. Spearman correlation was used to examine the association between insomnia severity and sleep quality on each subscale of psychological well-being and of sociodemographic and occupational variables. A significant contrast value was considered if *p* < 0.05.

## Results

### Sociodemographic and labor-related variables of prison guards

Forty-three guards participated in the present study, of whom 39 were male (90.6%) and 4 females (9.4%), with ages ranging from 19 to 42 years (M = 24.34, SD = 4.97). Most of the sample were single (90%) and had completed secondary education (62.7%). Sociodemographic information on the sample is shown in Table 1.

**Table 1:** Sociodemographic characteristics of the participants (*N* = 43).

Variable	*N*	%
**Gender**		
Masculine	39	90.6
Feminine	4	9.4
**Marital status**		
Single	39	90.6
Cohabitant	2	4.6
Married	1	2.4
Separated	1	2.4
**Level of education**		
Complete secondary education	27	62.7
Incomplete secondary education	1	2.4
Complete technical education	6	14
Incomplete technical education	1	2.4
Complete university education	3	6.9
Incomplete university education	5	11.6
**Age (years)**	M = 24.34	SD = 4.97

M, mean; SD, standard deviation. Percentages were calculated based on the total sample (*N* = 43).

Regarding labor-related variables, number of medical leaves in the last year, psychiatric or psychological support requirements, medication use, consumption of energy drinks, violence exposure at work by either colleagues of inmates, social support, total years of service at the institution and feeling cared for by the institution are all depicted in Table 2.

**Table 2:** Labor-related variables of the sample (*N* = 43).

**Variable**	*N*	%
**Have you considered seeing a psychologist or psychiatrist in the last year?**		
Yes	16	37.2
No	27	62.8
**Are you taking any medication?**		
Yes	3	6.9
No	40	93.1
**Have you been to the doctor because of an illness?**		
Yes	7	16.3
No	36	83.7
**Have you experienced violence at work in the last year from colleagues or prisoners?**		
Yes	8	18.6
No	35	81.4
**Do you drink energy drinks like Monster or Red Bull?**		
Yes	23	53.5
No	20	46.5
**Do you have someone you can call when you need to?**		
Yes	42	97.7
No	1	2.3
**Do you feel cared for by the institution?**		
Yes	17	39.5
No	26	60.5
**Number of sick leaves issued in the past year**		
0 leaves	18	41.9
1 leave	11	25.6
2 or more leaves	14	32.5
**Continuous variables**	***N***	
**Length of service at the institution (years)**		
Up to 1 year	16	37.2
1 to 3 years	14	32.6
3 to 6 years	9	20.9
10 or more years	4	9.3

### Psychological well-being of prison guards

Psychological well-being, assessed with the Ryff Psychological Wellbeing Scale, was moderate overall (M = 59.9, SD = 9.17), The lowest scores on this instrument were obtained on the environmental mastery and personal growth subscales (7.44 and 7.93, respectively). On the other hand, the highest scores were observed on the life purpose and positive relationships with others subscales (9.60 and 11.2, respectively). Scores between 1 and 7 indicate low levels of psychological well-being, whilst scores between 8 and 14 indicate moderate levels. No scores above 15 were obtained, so no cases of high psychological well-being were observed (Table 3).

**Table 3:** Descriptive statistics for each subscale of the PWB.

**Subscale**	M (SD)
Self-acceptance	9.23 (2.39)
Purpose in life	9.60 (3.65)
Environmental mastery	7.44 (2.36)
Positive relationships with others	11.2 (3.63)
Personal growth	7.93 (2.09)
Autonomy	9.44 (2.40)

M, mean; SD, standard deviation; PWB, psychological well-being scale.

### Insomnia severity levels and subjective sleep quality of prison guards

A descriptive analysis of the Insomnia Severity Index (ISI) indicates that the sample exhibits moderate levels of insomnia. The average total score for the sample was 17.87 (SD = 0.91), indicating moderate clinical insomnia, suggesting a high prevalence of clinically significant sleep difficulties and associated daytime impairment. Scores were interpreted as follows: 0–7 indicated no clinically significant insomnia, 8–14 subthreshold insomnia, (15–21) moderate insomnia, and (22–28) severe insomnia (21). In the current sample, ISI total score levels were: Subthreshold insomnia (8–14) was found in 7 guards (16.3%).

Moderate clinical insomnia 15–21 was observed in 29 guards (67.4%), and severe clinical insomnia 22–28, was present in 7 guards (16.3%). Clinically significant insomnia (Moderate + Severe) ≥15 was present in a total of 36 guards, which consists on 83.7% of the sample.

Sleep-related difficulties are consistent across different dimensions of the instrument; 55.8% of participants reported moderate to high or extremely high levels of difficulty falling asleep (25.6% and 2.3%, respectively). In the item staying asleep, 48.8% of the sample reported moderate levels of difficulty, whereas 18.6% reported high levels and 7% extremely difficult levels. Regarding waking up very early, 41.9% of the sample reported moderate levels, 20.9% severe levels and 11.6% very severe levels. Furthermore, a high proportion of the sample expressed dissatisfaction with their sleep patterns, stating that they were dissatisfied (48.8%) or very dissatisfied (30.2%), with 20.9% reporting a moderate satisfaction with sleep. As for the functional impact of their sleep problems, the results show that more than 60% of the sample felt that their sleep problem interfered a lot or very much with their daily functioning (39.5% and 30.2%, respectively), and 76.8% of the sample responded that their sleep problems were very noticeable to others. More than half of the guards reported high or very high levels of concern regarding their sleep problem (32.6% and 20.9%, respectively). Mean values are depicted in Table 4.

**Table 4:** Descriptive statistics for the items of the insomnia severity index (ISI).

Item ISI	M (SD)
Difficulty falling asleep	2.05 (0.89)
Difficulty staying asleep	1.98 (1.01)
Waking up very early	2.14 (1.04)
Current sleep satisfaction	3.09 (0.71)
To what extent do you think your sleep problem affects your day-to-day functioning?	2.98 (0.83)
How noticeable do you think your sleep problems are to others, in terms of how they affect your quality of life?	3.07 (0.85)
How concerned are you at the moment about your sleep problem?	2.56 (1.05)

M, Mean; SD, Standard Deviation.

Regarding subjective sleep quantity, the sample reported sleeping an average of 2.75 h per night whilst on duty and an average of 6.8 h per night when off duty (SD 94.4 and 130, respectively, calculated in minutes). Regarding how many days a week they woke up feeling rested, the average was 1.46 days with a SD of 1.40. In all cases, prison guards who had been with the institution for the longest (more than 3 years) reported sleeping less and feeling less rest. Subjective sleep quality was rated as fair by 37.2% of the sample, poor by 37.2% of the sample, and very poor by 25.6%.

### Correlation between variables

Correlation analyses between the total ISI score and psychological well-being (Ryff scale total scores) did not reveal a significant association (*p*-value = 0.974). Similarly, no significant relationships were observed between the ISI and any of the Ryff subscales.

When sociodemographic and occupational variables were included in the analysis, a significant positive correlation was observed between ISI and length of service (*p*-value = 0.023, *r* = 0.349), suggesting that longer service is associated with higher levels of insomnia. In contrast, overall psychological well-being showed no significant associations with these variables.

## Discussion

This study examined psychological well-being and insomnia among prison guards (gendarmes) in Valparaíso, Chile, revealing a concerning occupational health profile characterized by moderate clinical insomnia and compromised psychological well-being. The findings contribute to the limited literature on correctional officer health in Latin America and underscore the urgent need for targeted occupational health interventions in this high-stress profession.

### Psychological well-being in prison context: interpretation of Ryff PWB scores

The psychological well-being scores observed in our sample of Chilean prison guards warrant careful interpretation considering normative data from comparable populations. Using the 18-item Ryff Psychological WellBeing Scale, our participants reported a mean total psychological well-being score of 59.9 and a SD of 9.17.

Mean scores across six dimensions were: self-acceptance (9.23, SD = 2.39), purpose in life (9.60, SD = 3.65), environmental mastery (7.44, SD = 2.36), positive relationships with others (11.2, SD = 3.63), personal growth (7.93, SD = 2.09), and autonomy (9.44, SD = 2.40). A relevant comparison emerges from occupational health contexts. Delgado et al. (36) reported a mean total psychological well-being score of 85.38 using the 18-item Ryff scale among 482 registered nurses working in mental health settings. Assuming equal weighting across six subscales, this translates to approximately 14.23 per subscale, still considerably higher than our participants' scores. Saka et al. (37) examined Federal Road Safety Corps members in Nigeria, reporting mean total scores of 72.66 (SD = 10.33) for males and 73.54 (SD = 10.55) for females, which translates to approximately 12.11–12.26 per subscale. Even in this high-stress occupational context, our Chilean prison guards demonstrated lower psychological well-being, particularly in environmental mastery and personal growth.

The particularly low scores in environmental mastery (7.44) and personal growth (7.93) were significant. Environmental mastery reflects an individual's sense of competence in managing their environment and daily affairs, while personal growth captures feelings of continued development and self-realization (33). The correctional environment is characterized by unpredictability, limited autonomy, and exposure to potentially violent and traumatic events, all factors that directly undermine environmental mastery. Trounson et al. (38) noted that correctional officers face heightened risk of negative stress-related health conditions in their challenging work environment, which aligns with our findings of compromised environmental mastery. The low personal growth scores may reflect the repetitive, constrained nature of correctional work, where opportunities for professional development and self-actualization are limited.

Positive relationships with others (11.2) emerged as the highest-scoring dimension, though still below normative values. This finding suggests that interpersonal connections may serve as a protective factor for this sample. However, the substantial standard deviation (3.63) indicates considerable variability, with some officers experiencing significant social isolation. The moderate scores in self-acceptance (9.23), purpose in life (9.60), and autonomy (9.44) suggest that while guards maintain some sense of identity and meaning, these dimensions are also compromised.

### Insomnia and sleep disturbance in correctional officers: severity and prevalence

The prevalence of clinically significant insomnia observed in the present study appears higher than that reported among correctional workers in previous research. In the current sample, ISI total score levels were: Subthreshold insomnia (8–14) was found in 7 guards (16.3%).

Moderate clinical insomnia (15–21) was observed in 29 guards (67.4%), and Severe clinical insomnia (22–28), was present in 7 guards (16.3%). Clinically significant insomnia (Moderate + Severe) ≥15 was present in a total of 36 guards, which consists on 83.7% of the sample. In comparison, Ricciardelli et al. (26), reported that 64.9% of correctional workers screened positive for clinically significant insomnia symptoms, alongside reduced sleep duration, with workers sleeping an average of 6.0 h per night during work shifts and 7.2 h on off-shift nights, and reporting only 2.6 days per week waking feeling rested.

While this represents a high prevalence, our Chilean sample demonstrated higher insomnia symptoms and even more restricted sleep—averaging only 2.75 h per night on duty and 6.8 h off duty, with participants feeling rested only 1.4 days per week. The subjective sleep quality ratings in our sample were particularly poor: 37.2% rated their sleep as fair, 37.2% as poor, and 25.6% as very poor, with zero participants rating their sleep as good or excellent.

The specific insomnia symptoms reported by our participants paint a comprehensive picture of sleep dysfunction. More than half (55.8%) reported moderate-to-high difficulty falling asleep, while 74.4% experienced moderate-to-extreme difficulty staying asleep, and 73.5% reported moderate-to-very-severe difficulty waking too early. These findings suggest that our participants experience insomnia across all three temporal phases: sleep onset, sleep maintenance, and early morning awakening. The functional consequences were equally severe: 78.8% reported being dissatisfied or very dissatisfied with their sleep, more than 60% indicated that sleep difficulties interfered significantly with daily life, 76.8% reported that sleep problems were very noticeable to others, and more than 50% expressed high or very high concern about their sleep quality. The occupational context of Chilean prison guards likely contributes to this progressive deterioration. The 24-h shift schedules common in Chilean correctional facilities require officers to remain vigilant during extended periods, often with minimal opportunity for restorative sleep. The average of 2.75 h of sleep per night while on duty represents severe acute sleep deprivation, which accumulates over repeated shift cycles. Even the 6.8 h of sleep on off-duty nights falls short of the recommended 7–9 h for adults (39), suggesting that officers never fully recover from on-duty sleep loss.

Although differences in measurement procedures, occupational contexts, and sample characteristics should be considered, both studies indicate that sleep disturbances are highly prevalent among correctional personnel. The higher proportion of guards exceeding clinical insomnia thresholds in the present study may reflect the cumulative impact of occupational stressors, limited recovery opportunities, and contextual factors specific to the Chilean correctional system, although further comparative research is required to examine these potential explanations.

### Relationship between insomnia and psychological well-being

Despite no significant relationships were observed between the ISI and any of the RYFF subscales, the co-occurrence of severe insomnia and compromised psychological well-being in our sample is consistent with extensive literature documenting bidirectional relationships between sleep disturbance and mental health. While our cross-sectional design precludes causal inference, theoretical and empirical literature suggests multiple pathways through which insomnia and psychological well-being may be mutually reinforcing.

Sleep disturbance is both a symptom and a risk factor for psychological distress. Chronic insomnia is associated with increased risk of depression, anxiety, and reduced quality of life (40). The cognitive and emotional dysregulation resulting from chronic sleep deprivation may directly undermine the dimensions of psychological well-being measured by the Ryff scale. For example, sleep deprivation impairs executive function, emotion regulation, and stress reactivity—capacities essential for environmental mastery and autonomy. Similarly, chronic fatigue and reduced cognitive capacity may limit opportunities for personal growth and undermine sense of purpose in life.

Conversely, low psychological well-being may contribute to insomnia through multiple mechanisms. Individuals with low environmental mastery may experience their work environment as uncontrollable and threatening, leading to hyperarousal and rumination that interfere with sleep initiation and maintenance. Low purpose in life and self-acceptance may contribute to existential anxiety and negative self-focused cognition, which are established maintaining factors for insomnia (41). The correctional environment, characterized by unpredictability, exposure to violence, and moral distress, may simultaneously undermine both sleep and psychological well-being through chronic activation of stress response systems.

Ricciardelli et al. (26) found that poor sleep quality among Canadian correctional workers was associated with work stress and mental health disorders, supporting the interconnection between occupational stress, sleep, and psychological health. Plant et al. (42) demonstrated that sleep disturbance predicted suicidal thoughts and behaviors among public safety officers, highlighting the serious mental health consequences of chronic sleep problems in this occupational group. The combination of severe insomnia (mean ISI = 20.7) and compromised psychological well-being across all Ryff dimensions in our sample suggests a synergistic deterioration in both sleep and mental health that warrants urgent intervention.

### Occupational and contextual factors

#### Energy drink consumption as maladaptive coping

The finding that 53.5% of participants reported daily energy drink consumption represents a concerning pattern of maladaptive coping with sleep deprivation and occupational demands. This prevalence is substantially higher than rates reported in general young adult populations and suggests that energy drinks have become normalized as a strategy for managing the fatigue and vigilance demands of correctional work.

Energy drink consumption is increasingly recognized as both a consequence of and contributor to sleep problems, creating a vicious cycle of sleep deprivation and stimulant dependence. Kaur et al. (43) found in a longitudinal cohort study that young adult males who changed from non-energy drink users to users experienced increased depression, anxiety, and stress scores over a 2-year period. The high caffeine content of energy drinks (typically 80–300 mg per serving) can disrupt sleep architecture, reduce total sleep time, and increase sleep latency—effects that may be particularly pronounced when consumed during night shifts or in the hours preceding sleep attempts (44).

Several studies have documented energy drink use as a coping mechanism for occupational stress and sleep deprivation. Chappelle et al. (45) found that 19–20% of intelligence operators consumed three or more caffeinated beverages daily, with operators at greater odds of reporting increased caffeine use due to insufficient sleep, exhaustion from long work hours, and need to sustain vigilance. Hardon (46) documented that energy drink consumption is widespread among occupational populations including nightlife workers and security guards, often as a coping mechanism for long hours and sleep deprivation, though users reported adverse effects including heart palpitations, urinary tract infections, heartburn, dizziness, and stomach ulcers.

In the context of Chilean prison guards working 24-h shifts with an average of only 2.75 h of sleep on duty, energy drinks may be perceived as necessary for maintaining alertness and safety. However, this reliance on stimulants likely exacerbates the underlying sleep problems, contributing to the severe insomnia documented in our sample. The combination of chronic sleep deprivation, high caffeine intake, and occupational stress may create a self-perpetuating cycle: inadequate sleep drives energy drink consumption, which further disrupts sleep architecture and circadian rhythms, leading to increased sleep debt and greater reliance on stimulants. This pattern is particularly concerning given the associations between energy drink consumption and mental health problems documented in young adults (47, 48), which may compound the already compromised psychological well-being observed in our sample. For future studies, the consumption of coffee/black tea should be assessed, since they contain important additional sources of stimulant (caffeine).

Finally, it is recommended to provide internal training on sleep hygiene and consumption of stimulating substances containing caffeine, especially for shift workers.

#### Institutional support and sick leave

The finding that 60.5% of participants reported low institutional support represents a critical organizational factor that may contribute to both the sleep and psychological well-being problems observed in this sample. Perceived organizational support (the extent to which employees believe their organization values their contributions and cares about their well-being) is a well-established predictor of occupational health outcomes (49). In high-stress occupations such as correctional work, institutional support may be particularly important for coping with occupational stressors.

The high rate of frequent sick leave (68.4%) and the fact that 37.2% of participants considered seeking psychological care, suggest that the health consequences of this occupational environment are severe and recognized by the officers. However, the gap between those considering psychological care (37.2%) and those reporting low institutional support (60.5%) suggests potential barriers to help-seeking, including stigma, lack of accessible services, or organizational cultures that discourage acknowledgment of psychological distress. Aboh et al. (50) reported that operational stress negatively influences psychological well-being among paramilitary officers, while coping strategies positively influence it, suggesting that organizational interventions to enhance support and promote adaptive coping could mitigate the adverse effects of occupational stress.

The low institutional support reported by Chilean prison guards may reflect broader systemic issues in correctional systems, including inadequate staffing, limited resources for officer wellness programs, and organizational cultures that prioritize security operations over employee health. The 24-h shift schedules, which contribute directly to sleep deprivation, may themselves reflect inadequate staffing levels that necessitate extended work periods. Without organizational commitment to officer health and well-being, individual-level interventions are unlikely to be sufficient to address the severe sleep and psychological health problems documented in this study.

### Public health and occupational health implications

Our findings reinforce the relevance of sleep health as a critical component of occupational well-being among correctional officers. The observed association between insomnia symptoms and work-related factors, including years of service, is consistent with evidence from other safety-critical occupations in which irregular schedules, sustained stress exposure, and limited recovery time contribute to impaired sleep quality and reduced functional capacity. Previous research among shift workers, security personnel, and other high-demand occupational groups has similarly demonstrated that sleep disturbances are closely related to psychological strain, fatigue, and diminished work ability. In this context, insomnia among correctional officers should not be considered solely an individual health problem, but rather as an outcome influenced by organizational and occupational conditions.

The findings of this study have significant implications for public health and occupational health policy in Chile and other Latin American countries with similar correctional systems. The universal prevalence of clinical insomnia and the compromised psychological well-being across most Ryff dimensions represent a public health crisis within this occupational group. These findings suggest that current working conditions in Chilean correctional facilities are incompatible with basic standards of occupational health and safety.

### Limitations

Several limitations of this study should be acknowledged. First, the small sample size (*n* = 43) limits statistical power and generalizability. While our sample represents a substantial proportion of guards at the participating facility in Valparaíso (33.8%), it is a self-selective sub-population and so it may not be representative of correctional officers across Chile or in other Latin American countries.

An additional limitation relates to the potential risk of participant identification. Although data were collected without direct identifiers, questionnaires were administered in person and the relatively small sample size, combined with detailed sociodemographic and occupational information, may have increased the likelihood of indirect identification. This may have influenced participants' responses, particularly on sensitive topics such as health status, psychological support, or medication use, potentially introducing social desirability or response bias.

Second, the cross-sectional design precludes causal inference regarding the relationships among insomnia, psychological well-being, energy drink consumption, and occupational factors. While our findings are consistent with theoretical models and longitudinal research from other populations, we cannot determine whether insomnia causes reduced psychological well-being, whether psychological distress contributes to insomnia, or whether both are consequences of occupational stressors. Longitudinal research following correctional officers from the beginning of their careers would provide valuable insights into the temporal dynamics of these relationships and the cumulative effects of correctional work on health.

Third, the sample was predominantly male (90.6%), reflecting the gender composition of correctional officers in Chile but limiting our ability to examine gender differences in sleep and psychological well-being. Research in other occupational contexts has documented gender differences in sleep quality, insomnia prevalence, and psychological well-being (51), but our sample size and gender imbalance precluded such analyses. Future research with larger, more gender-balanced samples would allow for examination of whether male and female correctional officers experience different patterns of occupational health problems or respond differently to occupational stressors in the prison context.

Fourth, the study relied on self-report measures, which are subject to recall bias and social desirability effects. While the ISI and Ryff PWB scales are well-validated instruments with established psychometric properties, objective measures of sleep (e.g., actigraphy or polysomnography) would provide more precise characterization of sleep duration, sleep efficiency, and sleep architecture. Moreover, results concerning sleep quantity (perceived total sleep time) are highly likely to be biased/underestimated by the guards due to sleep misperceptions. Sleep sensitive shift systems could be a helpful approach to ameliorate sleep problems, increase sleep quantity and reduce insomnia.

Similarly, physiological measures of stress (e.g., cortisol, heart rate variability) could complement self-report measures of psychological well-being.

Finally, we did not assess several potentially important variables, including history of mental health disorders, substance use beyond energy drinks, physical health conditions, family and social support outside of work, and specific occupational exposures (e.g., violence, traumatic events). These factors may confound or moderate the relationships observed in this study. Future research should employ more comprehensive assessment protocols to disentangle the complex web of factors influencing correctional officer health.

## Conclusion

This study documents a severe occupational health crisis among prison guards in Valparaíso, Chile, characterized by clinical insomnia, compromised psychological well-being, and high prevalence of maladaptive coping strategies. The mean ISI score of 17.87 places this sample among the most sleep-deprived occupational groups documented in the literature, while the low scores across all six Ryff psychological well-being dimensions indicate pervasive mental health concerns. The progressive worsening of insomnia with length of service, the high prevalence of daily energy drink consumption, and the low institutional support reported by most participants, paint a picture of an occupational environment that systematically undermines officer health and well-being.

These findings have urgent implications for occupational health policy and practice in Chilean correctional facilities. The current working conditions that allow for an average of only 2.75 h of sleep on duty are incompatible with basic principles of sleep science and occupational health. Comprehensive, multi-level interventions are needed to address this crisis, including evidence-based fatigue management programs, accessible mental health services, organizational culture change to enhance institutional support, and policy reforms to establish and enforce standards for shift length, rest periods, and staffing ratios.

The health and well-being of correctional officers is not only a matter of occupational justice but also has implications for institutional security, the welfare of incarcerated individuals, and public health more broadly. Correctional officers experiencing severe insomnia and psychological distress cannot perform their demanding and often dangerous work safely and effectively. Investing in correctional officer health is therefore essential for the functioning of the correctional system and the safety of all individuals within it. Future research should employ longitudinal designs, larger and more diverse samples, and comprehensive assessment protocols to further elucidate the occupational health challenges facing correctional officers and to evaluate the effectiveness of interventions designed to promote their health and well-being.

## Data Availability

The raw data supporting the conclusions of this article will be made available by the authors, without undue reservation.
