# Supplementary material for: Insomnia severity and multidimensional psychological well-being in Chilean correctional personnel: a cross-sectional study
Source: Front Public Health. 2026 Oct 26;14:1782308. doi: 10.3389/fpubh.2026.1782308 (PMC13746978; doi:10.3389/fpubh.2026.1782308)
Supplement: Supplementary file 1 [file Supplementary_File_1.docx]

Datos Sociodemográficos:

1. Indica tu **género**

a) Femenino

b) Masculino

c) Otro

d) Prefiero no decir

2. Indica tu **edad** en años (número): ________

3. ¿Cuál es tu **estado civil?**

a) Soltero
b) Casado
c) Viudo
d) Separado
e) Conviviente
f) Otro ¿Cuál? _________________________

4. ¿Cuál es tu **nivel de educación**?

a) Básica incompleta
b) Básica completa
c) Media	incompleta
d) Media completa
e) Técnica incompleta
f) Técnica completa
g) Universitaria incompleta
h) Universitaria completa
i) Ninguno

5. ¿**Cuánto tiempo** llevas trabajando en Gendarmería?

__ años ___ meses

6. ¿Cuántas **licencias médicas** has solicitado el último año? ¿Cuál fue la causa?

7. ¿Has pensado o has ido al **psicólogo o psiquiatra en el último año**?

a) Sí

b) No

8. ¿Consumes alguna **medicación**? (si no recuerdas el nombre escribe para qué la tomas)

a) Sí,

b) No

9. ¿Has tenido que ir al médico general por una **enfermedad en el último año**? ¿Cuál?

a) Sí

b) No

10. ¿Has sufrido **violencia** en tu trabajo en el último año de parte de colegas o presos?

a) Sí,

b) No

11. ¿Consumes alguna **bebida energética tipo Monster o Red Bull**? ¿Cuantas por día?

a) Sí,

b) No

12. ¿Tienes **a alguien a quien llamar cuando lo necesitas**? (Familia, Amigos, Pareja, etc.)

a) Sí,

b) No

13. ¿**Te sientes cuidado** por la institución en la que trabajas?

a) Sí,

b) No

¿Qué podría hacer la institución para cuidarte más?

A continuación, se le presentarán una serie de afirmaciones. Por favor léalas y **marque con una sola “X” cada casilla que va del 1 al 7**. Donde **1 es “Totalmente de acuerdo”**; 2 “Algo de acuerdo”; 3 “Un poco de acuerdo; **4 “Neutro**; 5 “Un poco en desacuerdo”; 6 “Algo en desacuerdo”; y **7 es “Totalmente en desacuerdo”**:

|  | **1**  **Totalmente de acuerdo** | **2**  **Algo de acuerdo** | **3**  **Un poco de acuerdo** | **4**  **Neutro** | **5**  **Un poco en desacuerdo** | **6**  **Algo en desacuerdo** | **7**  **Totalmente en Desacuerdo** |
| --- | --- | --- | --- | --- | --- | --- | --- |
| 1. “**Me gusta la mayor parte de mi personalidad**” |  |  |  |  |  |  |  |
| 2. “Cuando miro la historia de mi vida, **estoy satisfecho de cómo han resultado las cosas** hasta ahora" |  |  |  |  |  |  |  |
| 3. “Algunas personas andan **sin rumbo** por la vida, pero **yo no soy una de ellas**” |  |  |  |  |  |  |  |
| 4. “Las **exigencias** de la vida cotidiana con frecuencia **me deprimen**” |  |  |  |  |  |  |  |
| 5. “En muchos sentidos, **me siento decepcionado por mis logros en la vida**” |  |  |  |  |  |  |  |
| 6. “Mantener relaciones cercanas ha sido **difícil y frustrante para mí**” |  |  |  |  |  |  |  |
| 7. “Vivo la vida un día a la vez y **no pienso en el futuro**” |  |  |  |  |  |  |  |
| 8. “En general, siento que **soy responsable de la situación en la que me encuentro**” |  |  |  |  |  |  |  |
| 9. “Soy **bueno** en el manejo de **mis responsabilidades diarias**” | **1**  **Totalmente de acuerdo** | **2**  **Algo de acuerdo** | **3**  **Un poco de acuerdo** | **4**  **Neutro** | **5**  **Un poco en desacuerdo** | **6**  **Algo en desacuerdo** | **7 Totalmente en Desacuerdo** |
| 10. “A veces **siento que he hecho todo lo que se debe hacer en la vida**” |  |  |  |  |  |  |  |
| 11. “Para mí, la vida ha sido un **proceso continuo de aprendizaje, cambio y crecimiento**” |  |  |  |  |  |  |  |
| 12. “Creo que es importante tener **nuevas experiencias** que desafíen mi forma de pensar sobre mí mismo y el mundo” |  |  |  |  |  |  |  |
| 13. “La gente me describiría como una persona **generosa, dispuesta a compartir mi tiempo con otros**” |  |  |  |  |  |  |  |
| 14. “Hace tiempo **dejé de intentar hacer grandes cambios o mejoras en mi vida**” |  |  |  |  |  |  |  |
| 15. “Tiendo a ser **influenciado** por personas con opiniones fuertes” |  |  |  |  |  |  |  |
| 16. “**No** he experimentado muchas relaciones **cercanas y de confianza** con los demás” | **1**  **Totalmente de acuerdo** | **2**  **Algo de acuerdo** | **3**  **Un poco de acuerdo** | **4**  **Neutro** | **5**  **Un poco en desacuerdo** | **6**  **Algo en desacuerdo** | **7 Totalmente en Desacuerdo** |
| 17. “Tengo **confianza en mis propias opiniones**, incluso si son diferentes a la forma de pensar de la mayoría de las otras personas" |  |  |  |  |  |  |  |
| 18. “Me juzgo a mí mismo por lo que creo que es **importante para mí**, no por lo que otros piensan que es importante” |  |  |  |  |  |  |  |
