# Supplementary material for: Insomnia severity and multidimensional psychological well-being in Chilean correctional personnel: a cross-sectional study
Source: Front Public Health. 2026 Oct 26;14:1782308. doi: 10.3389/fpubh.2026.1782308 (PMC13746978; doi:10.3389/fpubh.2026.1782308)
Supplement: Supplementary file 2 [file Supplementary_File_2.docx]

**Estudio sobre calidad del sueño y bienestar en personal de Gendarmería**

**Procedimiento:** Esta investigación estudia la calidad del sueño y el bienestar del personal de Gendarmería.

**Su participación:** Consiste en responder algunas preguntas sociodemográficas y dos encuestas breves. Las respuestas se tratarán de forma ANÓNIMA y CONFIDENCIAL, únicamente con fines de investigación

**Confidencialidad:** Toda opinión o información que Ud. nos entregue será tratada de manera **confidencial.** Nunca revelaremos su identidad. En las presentaciones que se hagan sobre los resultados de esta investigación no usaremos su nombre o apellido, ni tampoco revelaremos detalles suyos. Estos datos se utilizarán exclusivamente para fines académicos.

**Consentimiento:** Declaro que he leído el presente documento, se me ha explicado en qué consiste esta investigación y mi participación en la misma. Además, he tenido la posibilidad de aclarar mis dudas y tomo libremente la decisión de participar en el estudio a cargo de Isabel Benjumeda de la Universidad Adolfo Ibáñez.

**Firma:**
