# Supplementary material for: Insomnia severity and multidimensional psychological well-being in Chilean correctional personnel: a cross-sectional study
Source: Front Public Health. 2026 Oct 26;14:1782308. doi: 10.3389/fpubh.2026.1782308 (PMC13746978; doi:10.3389/fpubh.2026.1782308)
Supplement: Supplementary file 3 [file Supplementary_File_3.docx]

**ESTUDIO SOBRE INSOMNIO Y CALIDAD DEL SUEÑO**:

**PREGUNTAS SUBJETIVAS SOBRE CALIDAD DEL SUEÑO**

1- Número de horas de sueño totales por noche cuando estás de turno:
2- Número de horas de sueño totales por noche cuando **no** estás de turno:
3- Número de días por semana en los que te sientes descansado:
4- ¿Como CALIFICARÍAS TU CALIDAD DE SUEÑO?

| MUY MALA  0 | MALA  1 | REGULAR  2 | BUENA  3 | MUY BUENA  4 |
| --- | --- | --- | --- | --- |

**GRAVEDAD ACTUAL DEL PROBLEMA DE SUEÑO (ÚLTIMO MES)**:

|  | NADA | LEVE | MODERADO | GRAVE | MUY GRAVE |
| --- | --- | --- | --- | --- | --- |
| DIFICULTAD PARA **QUEDARSE** DORMIDO: | 0 | 1 | 2 | 3 | 4 |
| DIFICULTAD PARA **PERMANECER** DORMIDO: | 0 | 1 | 2 | 3 | 4 |
| DESPERTARSE **MUY TEMPRANO**: | 0 | 1 | 2 | 3 | 4 |

**SATISFACCIÓN ACTUAL CON EL SUEÑO:**

| MUY SATISFECHO  0 | SATISFECHO  1 | MODERADO  2 | INSATISFECHO  3 | MUY INSATISFECHO  4 |
| --- | --- | --- | --- | --- |

**INTERFERENCIA DEL PROBLEMA DE SUEÑO EN EL FUNCIONAMIENTO DIARIO**

| NADA  0 | POCO  1 | ALGO  2 | MUCHO  3 | MUCHÍSIMO  4 |
| --- | --- | --- | --- | --- |

**PERCEPCIÓN DE CUÁNTO NOTAS OTRAS PERSONAS EL PROBLEMA DE SUEÑO**

| NADA  0 | POCO  1 | ALGO  2 | MUCHO  3 | MUCHÍSIMO  4 |
| --- | --- | --- | --- | --- |

**¿QUÉ TAN PREOCUPADO ESTÁS ACTUALMENTE POR TU PROBLEMA DE SUEÑO?**

| NADA  0 | POCO  1 | ALGO  2 | MUCHO  3 | MUCHÍSIMO  4 |
| --- | --- | --- | --- | --- |
